# Clinical and Imaging Findings in 504 Cases With Left Ventricular Pseudoaneurysm Following Myocardial Infarction: A Comprehensive Systematic Review and Meta‐Analysis

**DOI:** 10.1155/crp/9208154

**Published:** 2026-08-17

**Authors:** Elmira Jafari Afshar, Amirhossein Tayebi, Parham Samimisedeh, Vahid Shahnavaz, Aryan Madady, Hadith Rastad, Neda Shafiabadi Hassani

**Affiliations:** ^1^ Cardiovascular Research Center, Alborz University of Medical Sciences, Karaj, Iran, abzums.ac.ir; ^2^ Herrington Heart and Vascular Institute, University Hospitals Cleveland Medical Center, Cleveland, Ohio, USA, uhhospitals.org

**Keywords:** clinical presentations, imaging findings, left ventricular aneurysm, left ventricular pseudoaneurysm, myocardial infarction

## Abstract

**Background:**

Left ventricular pseudoaneurysm (LVPA) is a rare but life‐threatening complication of myocardial infarction (MI). We pooled clinical and imaging findings of post‐MI LVPA reported in case reports/series and compared them with findings in post‐MI left ventricular aneurysm (LVA).

**Method:**

We comprehensively searched the literature published since 1991 on online databases. Individual‐level patient data (IPD) and aggregated‐level data (AD) studies were combined through a two‐stage meta‐analysis method.

**Results:**

We identified 379 eligible articles on LVPA (*N* = 504 patients). LVPA patients were predominantly male (70.4%) with a mean age of 65.4 years (95% CI: 62.4, 68.4). The time interval between the last MI and the diagnosis of LVPA varied from one day to 20 years; 54.5% fell into the acute/subacute category. On echocardiography, the most frequent locations of LVPA were posterior (34.5%) and inferior (21.2%). The average dimensions were 5.7 and 7.3 cm. On MRI, late gadolinium enhancement, primarily pericardial, was observed in 84% of patients. Arrhythmia was more frequently observed in LVA than LVPA (33.8% vs. 1%). Echocardiography showed a lower diagnostic value in LVPA than LVA (sensitivity: 81.4% vs. 97.5%). A higher percentage of LVPA than the LVA group died during hospitalization (13.8% vs. 4.7%) or after discharge (17.5% vs. 9.0%).

**Conclusion:**

LVPA is probably located on the posterior and inferior segments in most patients, contrary to LVA, which mainly involves the anterior and apical segments. Arrhythmia is likely a common clinical finding in LVA but not LVPA. Echocardiography may not always detect LVPA in 20% of patients.

## 1. Introduction

**FIGURE 1:**
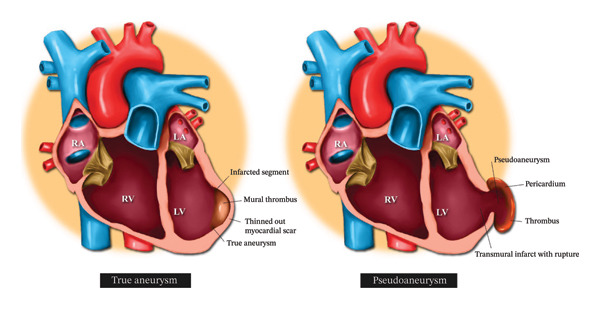
Schematic comparison of left ventricular true aneurysm and pseudoaneurysm after myocardial infarction. A true aneurysm consists of thinned scarred myocardium, whereas a pseudoaneurysm results from contained ventricular free‐wall rupture by the pericardium.

Patients with myocardial infarction (MI) may develop left ventricular aneurysms (LVAs) during the acute phase or later. These aneurysms are typically true LVAs, which form due to an expansion of the thinned myocardium and contain residual elements of the myocardial wall. However, in rare cases, they may be caused by the localized rupture of an infarcted area, resulting in a false left ventricular pseudoaneurysm (LVPA). As the wall of LVPA is made up of thrombus and/or pericardium without myocardial fibers (Figure 1), it is a more life‐threatening entity, yet less known, than LVA [1–3]. Proper differentiation between these two conditions is crucial for appropriate management. While LVA is often treated with elective surgery or medical treatment, LVPA typically requires urgent surgical intervention [4, 5].

While early diagnosis of LVPA and timely management are essential in reducing its mortality rate [6], our understanding of its clinical profile and imaging features is still limited and mainly comes from published case reports or case series. Also, to our knowledge, no previous review study has compared clinical characteristics and imaging features between LVPA and LVA. Therefore, in this comprehensive systematic review study, we aimed to provide a better understanding of the clinical and imaging features of LVPA by pooling data from post‐MI cases reported since 1991. We also compared these findings between our pooled cohorts of LVPA and LVA cases to help physicians distinguish between these two entities.

## 2. Methods and Materials

Our study was conducted according to the Preferred Reporting Items for Systematic Reviews and Meta‐analyses (PRISMA) guidelines [7]. As a systematic review and meta‐analysis of available literature, ethics committee approval was not required.

We included all eligible observational studies that reported imaging and clinical findings in patients with LVPA after MI. To compare these findings with those of the LVA, we also identified all studies with representative samples of patients with LVA following MI.

### 2.1. Search Strategy

We systematically searched online databases, including PubMed and Scopus, with an additional manual search in Google Scholar on September 1st, 2022, using the combination of relevant keywords in two domains:

1. False (and True) ventricular aneurysm
2. Myocardial infarction

Keywords from each domain were combined using the Boolean operator “OR,” and domains were connected with the “AND” Boolean operator.

Furthermore, we also manually screened the reference list of included and similar articles for possible additional citations. All citations retrieved from databases were imported into EndNote X9 software (version EndNote X9.3.2, Captivate Analytics, California, USA), and then duplicates were removed.

We presented the detailed search string of each database in Supporting Tables 1 and 2.

### 2.2. Study Inclusion

The title, abstract, and full text of imported studies were screened independently by two of our researchers (EJA & PS). Eligible studies that met our inclusion criteria were included for review and meta‐analysis. A third senior researcher (HR or NSH) resolved any disagreement between the researchers.

### 2.3. Selection Criteria

Studies that met all of the following eligibility criteria were included in our review study:

1. Written in English.
2. Included patients with post‐MI LVPA [or LVA for the comparison group].
3. Observational study design, including case report, case series, or cohort studies.

### 2.4. Data Extraction

Four researchers extracted the data from full‐text articles into a data extraction form in Microsoft Excel (Version 2016, Microsoft Corp., Redmond, WA, USA). We extracted the following data:

The first author’s name, country, year, study design, sample size, age, sex, types of symptoms, the time interval between the last MI and diagnosis of the false (and true) aneurysm, cardiovascular disease risk factors and comorbidities, electrocardiogram (ECG) (including ST elevation, pathologic Q waves, ventricular tachycardia, and normal ECG), imaging findings (including chest X‐ray, echocardiogram, computed tomography (CT) scan, and/or cardiac magnetic resonance imaging [CMR]), treatment strategies, follow‐up duration, and clinical outcomes were also extracted from the included studies.

Cases were categorized as acute (< 14 days), subacute (14–90 days), and chronic (> 90 days). As no universally accepted LV pseudoaneurysm‐specific temporal classification exists, these intervals were used as clinically pragmatic definitions reflecting the known timing of post‐MI mechanical complications and ventricular remodeling [6, 8, 9].

### 2.5. Risk of Bias Assessment

Two of our trained researchers assessed the quality of the included studies. Any disagreements were resolved with discussion. We used the Joanna Briggs Institute (JBI) critical appraisal tool to appraise the quality of the included studies. JBI’s critical appraisal tool contained 10 items; its total score ranges from 0 to 10 [10].

### 2.6. Statistical Analyses

To conduct our comprehensive systematic review study, we utilized a two‐step approach to pool relevant data. First, we combined individual‐level patient data (IPD) from case reports and case series into a single structured case series. These individual‐level data were subsequently transformed into study‐level summaries to ensure compatibility with aggregate data. Specifically, categorical variables were converted into event counts and corresponding sample sizes, while continuous variables were summarized as means and standard deviations when sufficient data were available. Then, we pooled these data with aggregate‐level data (AD) from other studies using a standard meta‐analysis method. In this second stage, study‐level summaries derived from IPD were pooled together with aggregate data from other studies using a random‐effects meta‐analysis model. This approach preserved the study as the unit of analysis, allowed appropriate weighting of each dataset within the pooled estimates, and accounted for both within‐study and between‐study variability. The use of a random‐effects framework was particularly important given the anticipated clinical and methodological heterogeneity across studies, including differences in study design, patient characteristics, imaging modalities, and data sources (IPD versus AD). This approach preserved the study as the unit of analysis and allowed appropriate weighting of each dataset within the pooled estimates.

We pooled categorical variables, including gender, symptoms, risk factors, history of previous surgery, treatment strategy, in‐hospital and follow‐up mortality, infarction site, and ECG results, as well as imaging results using meta‐analysis of single proportions. For continuous variables such as age, time from MI to diagnosis, follow‐up duration, and dimension size, we used a meta‐analysis of mean and standard deviation. To estimate the overall pooled proportion/mean and their 95% confidence intervals (CIs) in all meta‐analyses, we used a random‐effects model. All meta‐analyses were conducted using RStudio version 4.2.2 software.

Given the wide publication period and geographic diversity of the included studies, substantial clinical and methodological heterogeneity was anticipated, including differences in diagnostic techniques, patient characteristics, and management approaches. Therefore, all pooled analyses were conducted using random‐effects models to account for between‐study variability. Although heterogeneity statistics such as the *I* ^2^ metric are commonly reported, the predominance of single case reports and very small case series—often lacking within‐study variance estimates—limits the interpretability of these measures in our dataset.

## 3. Results

The detailed study selection process for LVPA and LVA is presented in Supporting Figures 1 and 2, respectively. After removing duplicates and ineligible articles, we included 379 eligible articles on post‐MI LVPA (*N* = 504 patients) [2, 11–168, 168–392] and 120 on post‐MI LVA (*n* = 20,968) [21, 24, 33, 39, 44, 46, 48, 49, 57, 62, 64, 65, 67, 73, 77, 83, 86, 89, 93, 99–101, 115, 121, 125, 135, 143, 144, 146, 147, 150, 154, 158, 160, 162, 167, 182, 184, 186, 194, 198, 211, 213, 218, 219, 225, 230, 231, 234, 237, 241, 245, 249, 255, 260, 261, 263, 272, 274, 286, 292, 294, 305, 308, 318, 326, 327, 329, 335, 338, 346, 355, 358, 360, 378, 382, 391–400].

Supporting Table 3 shows the quality of the included case series; all studies scored eight or higher.

### 3.1. Pseudoaneurysm

Characteristics of each included article are presented in Supporting Table 4. Regarding the study type, 366 were case reports, and 13 case series were published between 1991 and 2023. Cases were reported from 50 countries across the world; over 60% of them were reported from the USA (*n* = 105), Turkey (*n* = 63), and Italy (*n* = 56). Echocardiography, mainly TTE (transthoracic echocardiography), was the most frequently utilized imaging modality for diagnosis. Over 90% (464/504) of reported cases underwent surgical treatment.

Table 1 shows the demographic and clinical characteristics of reported cases. Based on our pooled analysis, patients were predominantly male [70.4% (95% CI: 65.8, 74.6)] with a mean age of 65.4 years (95% CI: 62.4, 68.4). They most frequently presented with congestive heart failure (CHF) symptoms [45.5% (95% CI: 24.7, 67.1, available data: *n* = 389)], followed by dyspnea [43.3% (95% CI: 24.4, 67.1, *n* = 357)], and chest pain [39.2% (95% CI: 24.0, 55.4, *n* = 409)]; with less frequency, cardiogenic shock [10.0% (95% CI: 0.4, 58.8, *n* = 352)], syncope [6.0% (95% CI: 3.5, 8.9, *n* = 340)], and arrhythmia [1.0% (95% CI: 0.0, 2.9, *n* = 241)] were also presented in these patients.

**TABLE 1:** Clinical characteristics of LVPA and LVA patients.

Variable		Pseudoaneurysm			Aneurysm		
		Valid sample size	Results		Valid sample size	Results	
Gender (male)		504	70.4 (65.8–74.6)		20,968	75.7 (69.3–81.6)	

Age (mean)		503	65.4 (62.4–68.4)		20,861	60.8 (58.9–62.8)	

Symptoms	Dyspnea	357	43.3 (24.4–63.2)		736	63.9 (38.8–85.7)	
	Chest pain	409	39.2 (24.0–55.4)		1857	56.9 (43.1–70.2)	
	CHF	389	45.5 (24.7–67.1)		19,514	59.7 (44.9–73.6)	
	Shock	352	10.5 (0.4–58.8)		17,714	6.1 (1.4–13.0)	
	Syncope	340	6.0 (3.5–8.9)		17,711	6.3 (1.4–13.5)	
	Arrhythmia	326	1.0 (0.0–2.9)		18,978	33.8 (22.1–46.0)	

Time from MI to diagnosis, mean	Acute	241	29.0% (23.4–35.2)	4.4 (3.3–4.9) days	29	20.7% (7.9)	5.5 (2.6–8.3) days
	Subacute		25.7% (20.3–31.7)	46.3 (40.1–52.6) days		55.2% (35.7–73.5)	39.4 (25.1–53.7) days
	Chronic		45.2% (38.8–51.7)	10.4 (8.9–12.1) months		24.1% (10.3–43.5)	15.3 (7.1–23.6) months
	Total (month)	329	5.1 (1.3–8.9)		18,235	27.8 (17.4–37.2)	

Risk factor	HTN	312	52.2 (21.8–81.9)		1649	58.8 (50.4–67.1)	
	DM	349	30.5 (16.9–45.7)		1993	27.5 (22.3–33.0)	
	Smoker	342	61.9 (24.2–93.5)		373	50.7 (28.9–72.4)	
	Hyperlipidemia	35	65.9 (229.6–94.9)		385	52.2 (19.4–84.0)	

Previous surgery	CABG	279	21.1 (16.5–26.4)		73	15.1 (7.7–25.3)	
	Valvular		3.9 (1.9–6.9)			2.7 (0.3–9.5)	
	Without surgery		69.9 (64.1–75.2)			82.2 (71.4–90.1)	

Treatment	Surgery	471	95.5 (84.2–100)		76	72.3 (60.9–82.0)	
	Conservative	341	20.8 (16.3–25.5)			26.3 (16.8–37.7)	
	Intervention		6.5 (4.1–9.6)			1.3 (0.03–7.1)	

Hospital death		384	13.8 (9.9–18.1)		18,204	4.7 (2.9–6.8)	

Follow up	Follow‐up duration mean (month)	88	22.1 (5.7–38.3)		647	47.3 (29.2–65.4)	
	Follow‐up death	123	17.5 (10.4–25.7)		647	9.0 (4.8–14.1)	

ECG	ST elevation	192	43.2 (36.1–50.5)		63	28.6 (17.8–41.3)	
	Pathologic Q waves		42.7 (35.6–50.0)			61.9 (48.8–73.9)	
	Tachycardia		9.4 (5.6–14.4)			22.2 (12.7–34.5)	
	Normal		3.6 (0.1–7.3)			6.3 (1.7–15.4)	

Infarction site	Inferior	490	37.0 (21.8–53.5)		18,063	11.9 (6.2–19.0)	
	Anterior		20.1 (11.6–29.8)		18,965	84.9 (66.8–97.0)	
	Lateral		9.0 (5.9–12.5)		759	3.9 (0.3–10.3)	
	Posterior		16.3 (5.3–31.0)		1091	4.2 (0.1–12.4)	

*Note:* Data are presented as percentages (%) and 95% confidence intervals.

Abbreviations: CABG, coronary artery bypass graft; CHF, congestive heart failure; DM, diabetes mellitus; HTN, hypertension; LVA, left ventricular aneurysm; LVPA, left ventricular pseudoaneurysm; MI, myocardial infarction; NA, not applicable; PA, pseudoaneurysm.

The time interval between the last MI and the diagnosis of LVPA varies from one day to 20 years (*n* = 329); the mean (95% CI) is 5.1 (1.3, 8.9) months; according to this time interval, 29.0% of patients fell into the acute category (0, 14 days), 25.7% into the subacute category (14, 90 days), and 45.5% into the chronic category (more than 90 days) (Table 1).

Previous cardiac surgery was reported in 33.3% (95% CI: 27.8, 39.2) of the cases (*n* = 279), including coronary artery bypass graft (CABG) (21.1%) and valvular surgery (3.9%). A history of smoking, hypertension (HTN), and diabetes mellitus (DM) was presented in 61.9% (24.2, 93.5), 52.2% (95% CI: 21.8, 81.9), and 30.5% (95% CI: 16.9, 45.7) of patients, respectively (Table 1).

The ECG data on admission were available for 192 cases, of whom 185 (96.3%) had an abnormal finding(s), mainly ST‐segment elevation (43.2% (95% CI: 36.1, 50.1)), pathologic Q waves [42.7% (95% CI: 35.6–50.0)], and/or tachycardia [9.4% (95% CI: 5.6, 14.4)]. The location of infarction was in order by frequency: inferior 37% (95% CI: 21.8, 53.5), anterior 20.1% (95% CI: 11.6, 29.8), posterior 16.3% (95% CI: 5.3, 31.0), and lateral in 9.0% (95% CI: 5.9, 12.5) (as determined by the presence of ST elevation or Q waves in different leads).

The imaging findings are shown in Table 2. Chest X‐ray findings were reported in 92 patients; abnormalities such as cardiomegaly, cardiac mass, and cardiac silhouette sign were observed in 60.4% (95% CI: 50.2, 70.6), 35.1% (95% CI: 25.1, 45.1), and 25.2% (95% CI: 16.1, 34.4) of cases, respectively (Table 2).

**TABLE 2:** Imaging findings of LVPA and LVA patients.

Diagnostic imaging			Pseudoaneurysm		Aneurysm	
			Valid sample size	Result	Valid sample size	Result
Chest X‐ray	Cardiomegaly		91	60.4 (50.2–70.6)	24	66.7 (44.7–84.4)
	Cardiac mass			35.1 (25.1–45.1)		20.8 (7.1–42.1)
	Cardiac silhouette sign			25.2 (16.1–34.4)		12.5 (2.6–32.3)
	Normal			9.7 (3.6–15.9)		12.5 (2.6–32.3)

Echocardiography	Diagnosis on Echo		328	81.4 (76.8–85.5)	80	97.5 (91.2–99.7)
	Type	TTE	328	81.7 (77.1–85.7)	79	87.3 (77.9–93.7)
		Doppler		13.1 (9.6–17.2)		1.2 (0.03–6.8)
		TEE		12.2 (8.8–16.2)		10.1 (4.4–18.9)
		Contrast echo		1.2 (0.3–3.1)		2.5 (0.03–8.8)
	Location of outpouching	Apical	329	13.8 (9.8–18.3)	1925	38.2 (11.9–71.1)
		Inferior		21.2 (3.0–47.2)	1925	1.1 (0.0–3.2)
		Anterior		17.1 (3.7–35.6)	1848	35.2 (8.7–67.9)
		Lateral		6.4 (1.5–13.5)	1807	0.7 (0.0–2.3)
		Posterior		34.5 (8.3–66.3)	1925	8.5 (3.6–14.9)
	Diametersmean (cm)	Maximal sac diameter (long axis) (cm)^∗^	103	5.7 (5.2–6.5)	25	8.3 (6.0–10.6)
		Maximal sac diameter (short axis) (cm)^∗^	37	7.3 (6.4–8.2)	19	6.2 (4.5–7.9)
		Neck diameter 1 (cm)^∗^	12	4.1 (2.7–5.3)	9	4.4 (3.2–5.6)
		Neck diameter 2 (cm)^∗^	7	2.5 (0.4–4.7)	NA	
	Clot presentation		66	92.4 (83.2–97.5)	80	90.0 (81.2–95.6)
	LVEF (mean)		209	36.5 (34.9–38.0)	2488	32.5 (29.7–35.2)

Cardiac magnetic resonance	Location	Apical	85	24.7 (15.9–35.2)	22	45.4 (24.4–67.8)
		Inferior		50.6 (39.5–61.6)		45.4 (24.4–67.8)
		Anterior		2.4 (0.2–8.2)		0
		Lateral		15.3 (8.4–24.7)		9.2 (1.1–2.9)
		Posterior		15.3 (8.4–24.7)		0
		Basal		7.1 (2.6–14.7)		0
	Diametersmean (cm)	Maximal sac diameter (long axis) (cm)^∗^	29	6.2 (3.9–8.2)	9	5.8 (2.3–9.5)
		Maximal sac diameter (short axis) (cm)^∗^	11	10.0 (7.0–11.0)	9	6.9 (4.8–9.1)
		Neck diameter 1 (cm)^∗^	4	3.0 (2.25–3.0)	NA	
		Neck diameter 2 (cm)^∗^	4	3.8 (1.9–5.4)	NA	
	Clot presentation		47	95.7 (85.4–99.5)	9	88.9 (51.7–99.7)
	LVEF (mean)		18	32.9 (28.2–37.6)	NA	
	Pericardial		25	84.0 (63.9–95.5)	5	0

LGE	Myocardial			16.0 (4.5–36.1)		80.0 (28.3–99.5)

CT scan	Location	Apical	114	24.5 (16.9–33.5)	19	42.1 (20.2–66.5)
		Inferior		28.1 (20.1–37.2)		26.3 (9.1–51.2)
		Posterior		23.7 (16.2–32.6)		10.5 (1.3–33.1)
		Lateral		19.3 (12.5–27.7)		15.8 (3.3–39.6)
		Anterior		9.6 (4.9–16.6)		5.2 (0.1–26.0)
		Basal		6.1 (2.5–12.2)		0
	Type of the CT	CT angiography	101	49.5 (39.4–59.6)	17	35.3 (14.2–61.7)
		Cardiac CT scan		36.6 (27.2–46.8)		52.9 (27.8–77.0)
		Chest CT scan		13.9 (7.7–22.1)		11.8 (1.4–36.4)
	Diametersmean (cm)	Maximal sac diameter (long axis) (cm)^∗^	55	6.9 (5.6–8.1)	11	6.7 (4.9–8.4)
		Maximal sac diameter (short axis) (cm)^∗^	23	12.1 (3.7–20.6)	11	5.6 (4.4–6.8)
		Neck diameter 1 (cm)^∗^	11	4.0 (2.1–6.1)	NA	
		Neck diameter 2 (cm)^∗^	7	2.8 (0.6–4.9)	NA	

*Note:* Data are presented as percentages (%) and 95% confidence intervals; cm, centimeter. LVPA, left ventricular pseudoaneurysm; PA, pseudoaneurysm;

Abbreviations: CT, computed tomography; LVA, left ventricular aneurysm; LVEF, left ventricular ejection fraction; MRI, magnetic resonance imaging; NA, not applicable; TEE, trans esophageal echocardiography; TTE, trans thoracic echocardiography.

^∗^Neck diameter and pseudoaneurysm sac dimensions are reported as provided in the original studies. When two measurements were available, they generally represented orthogonal long‐ and short‐axis diameters as reported by echocardiography, CCTA, or cardiac MRI.

Based on available data on echocardiography evaluations (*n* = 328), this imaging modality failed to diagnose LVPA in 18.6% (14.5, 23.2%) of cases. Based on available data on echocardiography evaluations (*n* = 328), LVPA was not identified on echocardiography in 18.6% (95% CI: 14.5%–23.2%) of cases. The most frequent location of LVPA was posterior [34.5% (CI 95%:8.3, 66.3)], followed by inferior [21.2 (95% CI: 0.3, 47.2)], and anterior [17.1% (95% CI: 3.7, 35.6)]. The mean (95% CI) left ventricular ejection fraction (LVEF) was 36.5% (34.9, 38.0) (*n* = 209). The mean (95% CI) of the two reported dimensions of the LVPA were 5.7 (95% CI: 5.2, 6.5, *n* = 103) and 7.3 (CI 95%: 6.4, 8.2) centimeters (Table 2).

Data on cardiac magnetic resonance were available in 85 patients. Common locations of the LVPA were inferior at 50.6% (CI 95%: 39.5, 61.6). The mean (95% CI) LVEF on MRI, reported in 18 patients, was 32.9% (CI 95%: 28.2, 37.6). Late gadolinium enhancement (LGE) was reported in 25/85 patients, of whom the majority had pericardial LGE [84.0% (CI 95%: 63.9, 95.5)] (Table 2).

A total of 114 cases underwent CT scans, mainly CT angiography (CTA) [49.5% (95% CI: 39.4, 59.6)]. The most prevalent location of LVPA in CT scans was inferior at 28.1% (95% CI: 20.1, 73.2), followed by apical at 24.5% (16.9, 33.5), and posterior at 23.7% (95% CI: 16.2, 32.6). Hospital death and follow‐up death occur in 13.8% (95% CI, 9.9–18.1) and 17.5% (95% CI, 10.4–25.7) of LVPA cases, respectively (Table 2).

### 3.2. Comparisons Between LVPA and LVA

In both LVPA and LVA groups, over 70% of the cases were male (70.4% vs. 75.7%, respectively), and their mean age fell in the seventh decade of life (65.4 vs. 60.8 years, respectively). The two groups were similar regarding the prevalence of cardiovascular disease risk factors, including HTN, DM, hyperlipidemia, and smoking (Table 1).

On admission, while one out of three patients with LVA presented with arrhythmia, only one out of 100 LVPA patients showed this symptom; however, cardiogenic shock was more prevalent among the LVPA than LVA group (10.0% vs. 6.1%, respectively). In addition, patients with LVPA less frequently had dyspnea (43.3% vs. 63.9%, respectively), chest pain (39.2% vs. 56.9%, respectively), and CHF symptoms (45.5% vs. 59.7%, respectively) in comparison with those in the LVA group (Table 1).

The mean time interval from MI to diagnosis was shorter in the LVPA group than in the LVA group (5.1 (95% CI 1.3–8.9) vs. 27.8 (95% CI 17.4–37.2) months, respectively). The most common previous cardiac surgery was CABG in both groups (Table 1).

In terms of ECG findings, the group with LVPA showed a higher frequency of ST‐segment elevation (43.2% compared to 28.6% in the LVA group). The LVPA group had a lower frequency of the presence of pathologic Q waves (42.2% compared to 61.9% in the LVA group). The most common location of infarction was inferior in the LVPA group (37% with a 95% CI of 21.8–53.5), while it was anterior in the LVA group (84.9% with a 95% CI of 66.8–97.0) (Table 1).

On chest X‐ray, a higher percentage of the LVPA group had cardiac mass (35.1% vs. 20.8%) but a lower percentage of them had evidence of cardiomegaly (60.4% vs. 66.7%) (Table 2).

Echocardiography had a lower sensitivity for the diagnosis of LVPA compared to the LVA group (81.4% vs. 97.5%). Compared with CT and CMR, echocardiography demonstrated a lower rate of LVPA detection. In contrast to LVPA, mainly located on posterior and inferior (34.5% and 24.2%, respectively) segments, LVA involved apical and anterior segments (38.2% and 35.2%, respectively). Patients with LVA had a higher mean (95% CI) LVEF than patients with LVPA [36.5% (34.9, 38.0) vs. 32.5% (29.7, 35.2)]. On cardiac MRI, approximately four out of five patients with LVPA (21/25) had a pericardial LGE, but none of the five patients in the LVA group had this pattern of LGE (Table 2).

The LVPA group, compared to the LVA group, had higher hospital mortality (13.8% vs. 4.7%, respectively) and follow‐up death (17.5% vs. 9.0%, respectively).

## 4. Discussion

This comprehensive pooled clinical and imaging data of all available studies describing LVPA after MI were compared with the findings in a pooled cohort of LVA patients.

Since 1991, a total of 504 cases of LVPA following MI have been reported worldwide. The majority of these cases were male, with ages ranging from 33 to 97 years old. In approximately half of the cases, LVPA was diagnosed 90 days or more after the last MI, indicating chronic LVPA. CHF symptoms were reported in nearly one‐third of the cases at presentation, and about 10% of them experienced cardiogenic shock upon admission. Additionally, just over 20% of the patients had a prior history of CABG. The most common abnormalities on ECG were STE and Q waves. Additionally, cardiomegaly and cardiac masses were commonly detected on CXR. Echocardiography was the primary diagnostic tool for approximately 80% of LVPA cases, with the most common locations being posterior, followed by inferior. The LVPA patients were roughly older than LVA patients, but gender was mainly male in both groups. Regarding clinical symptoms on admission, arrhythmia was common in the LVA group but not in LVPA. LVPA patients had a shorter mean time interval from the last MI to diagnosis compared to LVA patients. Contrary to LVPA, the most common location of LVA was apical and anterior. LVEF was a little higher in the LVPA group. ST‐segment elevation was observed less frequently in LVA cases, and cardiomegaly was the most common finding on CXR in these patients. Contrary to LVA, pericardial LGE was a common finding in LVPA patients on cardiac MRI. Hospital mortality was about three times higher in the LVPA group compared to the LVA group. About 95% and 73% of LVPA and LVA cases were treated surgically, respectively.

Gender patterns in LVPA and LVA were roughly the same and similar to MI; about 70% of MI cases are male. However, LVA patients were slightly older than the LVPA group [401, 402].

Post‐MI LVPA occurs when a pericardial adhesion restricts a true rupture of the ventricular wall. In contrast, LVA appears following cardiac remodeling, which leads to a change in geometry of the left ventricular cavity and the formation of LVA due to the alteration of muscle fiber cells [403]. Aging is associated with an increased risk of impaired ventricular remodeling, a possible mechanism involved in LVA. Elderly patients more frequently experience suppressed post‐MI inflammation, which is vital to prevent ventricular remodeling [404].

A higher rate of arrhythmia observed in LVA compared to LVPA can be attributed to their distinct etiologies. Structural remodeling in LVA leads to the formation of regions with slow conduction and conduction block, which increases the likelihood of arrhythmias. On the other hand, LVPA may not exhibit the same extent of structural remodeling, potentially explaining the lower incidence of arrhythmias in this condition [234, 405].

Furthermore, LVPA patients may have a higher risk of developing cardiogenic shock compared to LVA patients due to the increased risk of cardiac tamponade and ventricular rupture in LVPA. LVPA typically occurs more commonly in the acute phase of the infarction, while LVA is more commonly seen in the chronic phase. On ECG, LVPA is characterized mainly by ST‐segment elevation, while LVA is characterized by signs of an old MI, such as Q waves. LVPA is a life‐threatening emergency condition that more frequently results in worse outcomes compared to LVA. According to our pooled analysis, LVPA patients have a significantly higher mortality rate than LVA patients, with LVPA patients dying approximately 2–3 times more than their LVA counterparts during hospitalization and after discharge.

Still, early diagnosis can lead to timely management and improve the prognosis in the affected patients. In this way, the distinction between different outpouchings of the left ventricle, i.e., LVPA and LVA, is imperative for choosing a proper treatment strategy. While almost all patients, with or without symptoms, in the LVPA group undergo cardiac surgery, management strategy in LVA is largely determined based on the presence of symptoms [342].

Echocardiography is the most commonly used imaging modality for assessing structural heart diseases, including outpouchings of the left ventricle, due to its availability, cost‐effectiveness, and safety. However, our pooled analysis indicates that echocardiography has notable limitations in detecting LVPA, with approximately 20% of cases remaining unrecognized. Standard TTE may fail to adequately visualize the pseudoaneurysm cavity or its communication with the left ventricle. In this context, contrast‐enhanced echocardiography can serve as a valuable adjunct in selected cases. By improving left ventricular cavity opacification and enhancing endocardial border delineation, contrast agents may facilitate identification of the pseudoaneurysm sac and its narrow neck, thereby increasing diagnostic confidence—particularly in posteriorly located lesions or in patients with suboptimal acoustic windows [406].

In contrast, echocardiography showed better performance in diagnosing LVA [276]. Some echocardiography features, such as the location of observed outpouchings and neck diameters, could also help to distinguish LVPA from LVA; contrary to LVA, LVPA is more frequently located on the posterior and inferior segments. Their different locations of infarction could explain this difference [407]. However, another proposed explanation is that patients with anterior LVPA are at an increased risk of ventricular rupture leading to death before diagnosis [57].

Contrary to LVA, LVPA is usually characterized by a narrow neck on TTE echocardiography, which manifests as a turbulent flow on Doppler echocardiography [1, 355]; noteworthy, some chronic LVPA may have a wide base similar to LVA, causing misdiagnosis. However, most included studies failed to provide data on neck diameters in both groups.

MRI and CT scans, especially CT angiography, have higher accuracy than echocardiography for the diagnosis of cardiac outpouchings, including LVPA; though, in practice, their utilization is limited by factors such as less availability, no portability, and high costs [408].

Early and accurate imaging has important clinical implications because LVPA carries a substantially higher risk of rupture and mortality than LVA. When echocardiographic findings are inconclusive or discordant with clinical suspicion, prompt escalation to CMR or cardiac CT is warranted, as these modalities provide superior delineation of myocardial integrity and rupture tracts. Given the distinct prognostic and therapeutic implications of LVPA, timely diagnostic confirmation is essential and may directly influence decisions regarding urgent surgical evaluation [409, 410].

Current evidence suggests that pericardial LGE can be a valuable tool for differentiating LVPA from LVA. While LGE is present in the wall of the aneurysm sac in both LVPA and LVA on cardiac MRI, delayed enhancement of the pericardium is considered a characteristic feature of pseudoaneurysm. This finding indicates the presence of pericardial inflammation and fibrosis caused by blood leakage into the pericardial space following ventricular rupture [144, 285].

The primary aim of this study is to clarify the diagnostic features distinguishing LVPA from LVA rather than to evaluate therapeutic approaches. Although LVPA typically carries higher rupture risk and often prompts surgical consideration, our focus is on improving diagnostic accuracy and appropriate imaging escalation. By strengthening recognition and proper imaging selection, clinicians can make more informed decisions without this review extending beyond the available evidence into treatment recommendations.

### 4.1. Limitations and Strengths

This study has several limitations. Because the analysis relied on published reports, some variables—particularly imaging characteristics and clinical outcomes—were incompletely reported. The included studies spanned more than 3 decades and multiple countries with variable diagnostic and reporting standards, contributing to substantial heterogeneity. In addition, most LVPA data were derived from case reports and small case series, which are susceptible to selection and publication bias and limited the feasibility of formal heterogeneity or sensitivity analyses. Because a uniform reference standard was not consistently applied across reports, the observed proportions of LVPA detection by different imaging modalities should not be interpreted as formal estimates of diagnostic sensitivity. A further limitation is the substantial reliance on case reports and small case series within the LVPA cohort. These reports are inherently prone to selection and publication bias, as unusual or severe cases are more likely to be published, potentially influencing the observed clinical characteristics and management patterns. Given the heterogeneity and limited sample size of individual reports, formal sensitivity analyses were not feasible; therefore, the findings should be interpreted with caution regarding their generalizability. These factors also resulted in wide confidence intervals for several pooled estimates, reflecting limited statistical precision and small numbers of contributing studies or patients. As such, the proportions presented here should be understood as descriptive patterns rather than precise population estimates, and the evidence base for each analysis should be interpreted in light of the variable denominators across reported outcomes.

Despite these limitations, this study represents one of the most comprehensive pooled analyses of post‐MI LVPA to date. By systematically integrating data from numerous reports and comparing findings with a large pooled cohort of LVA patients, our analysis provides a broader characterization of the clinical and imaging features of LVPA and offers practical insights to assist clinicians in differentiating these two entities.

## 5. Conclusion

Our analysis indicates that LVPA most often arises in the posterior or inferior segments, whereas LVA typically involves the anterior and apical walls. Arrhythmias and ECG signs of prior infarction were more frequent in LVA, while LVPA more often showed persistent ST‐segment elevation and a new cardiac silhouette on chest radiography. Key imaging red flags for LVPA included a narrow neck‐to‐cavity ratio, myocardial wall discontinuity, and pericardial LGE on CMR. Echocardiography missed nearly one‐fifth of LVPA cases—particularly posterior lesions—highlighting the need for early CT or CMR when initial imaging is inconclusive. LVPA displays a consistent set of imaging characteristics that support earlier recognition, and these patterns offer practical guidance for distinguishing it from LVA and avoiding diagnostic delay.

## Funding

No funding was received for this study.

## Disclosure

A previous version of this manuscript was published as a preprint: E.J. Afshar, A. Tayebi, P. Samimisedeh, et al. Clinical characteristics and imaging findings of post‐infarction LVPA versus aneurysm: a pooled analysis of 21,472 patients. medRxiv (Preprint). 2023. https://doi.org/10.1101/2023.02.23.23286381.

## Conflicts of Interest

The authors declare no conflict of interest.

## Supporting Information

Additional supporting information can be found online in the Supporting Information section.

## Supporting information


**Supporting Information** The following Supporting Information is available with this manuscript: Supporting Table 1: Search strategy for left ventricular pseudoaneurysm. Supporting Table 2: Search strategy for left ventricular aneurysm. Supporting Figure 1: PRISMA flowchart diagram of study selection for pseudoaneurysm cases. Supporting Figure 2: PRISMA flowchart diagram of study selection for true aneurysm cases. Supporting Table 3: Quality assessment of included studies. Supporting Table 4: Summary of case series and cohort studies. These supporting files are provided as submitted by the authors and have not been modified during production.

## Data Availability

The data utilized for this pooled analysis were extracted from published articles, with each source properly cited for reference.
