# Supplementary material for: Clinical and Imaging Findings in 504 Cases With Left Ventricular Pseudoaneurysm Following Myocardial Infarction: A Comprehensive Systematic Review and Meta‐Analysis
Source: Cardiol Res Pract. 2026 Aug 17;2026:9208154. doi: 10.1155/crp/9208154 (PMC13479120; doi:10.1155/crp/9208154)
Supplement: Supplementary file 1 [file CRP-2026-9208154-s001.docx]

| **Research question**  **Supplementary. Table 1.** Search strategy for left ventricular pseudoaneurysm. | What are the clinical outcomes, prognosis, and imaging characteristics of the left ventricular pseudo aneurysm post-myocardial infarction? |
| --- | --- |
| **Research title** | Clinical characteristics and imaging findings in post-infarction left ventricular pseudo aneurysm and aneurysm, A pooled analysis of 21000 patients |
| **PICOT** | **P:** People with left ventricular pseudo-aneurysm post-myocardial infarction  **E:** NA  **C:** NA  **O:** Clinical outcomes, Strategies of treatment, Prognosis, different imaging modalities characteristics  **T:** Observational studies |
| **Keywords** | Pseudoaneurysm, LVPA, Myocardial infarction, Systematic review, Left ventricle rupture, Myocardial infarction complications, False aneurysm |
| **Search strategy**  (Searched on September 1^st^ 2022) | **Scopus:** ( ( TITLE-ABS-KEY ( "Cardiac magnetic resonance imaging" ) )  OR  ( TITLE-ABS-KEY ( "Echocardiography" ) )  OR  ( TITLE-ABS-KEY ( "Computed tomography" ) ) )  AND  ( ( TITLE-ABS-KEY ( "False aneurysm" ) )  OR  ( TITLE-ABS-KEY ( "Ventricular Pseudoaneurysm" ) ) )  **Total citations that were retrieved from Scopus: 3665** |
|  | **PubMed:** (((("Aneurysm, False"[Mesh])) OR ("Ventricular Pseudoaneurysm"[Title/Abstract])) OR ("False aneurysm"[Title/Abstract])) AND (((("Magnetic Resonance Imaging"[Mesh]) OR ("Computed Tomography Angiography"[Mesh])) OR ("Tomography, X-Ray Computed"[Mesh:NoExp])) OR ("Echocardiography"[Mesh]))  **Total citations that were retrieved from PubMed: 5545** |
|  | **Google Scholar:** Phrase searches: “left ventricular pseudoaneurysm”, “ventricular pseudoaneurysm”, “LV pseudoaneurysm”, “false aneurysm”, “contained myocardial rupture”, combined with “myocardial infarction”, “post‑MI”, “MI” |
|  | **The total number of included studies: 379 articles comprise 366 case reports and 13 case series** |
| **Search Limits** | **Date range:** January 1991 – September 1, 2022 |
|  | **Rationale for start year:** 1991 corresponds to the earliest modern imaging‑confirmed LVPA cases meeting criteria |
|  | **Language:** English |
|  | **Included:** Case reports, case series, cohort studies |
|  | **Excluded:** Reviews, conference abstracts, animal studies |
| **Screening Results** | **Number of total citations that were retrieved after Scopus and PubMed systematic search and google scholar manual search: 9210**  **Remained articles after duplicates removal: 7630**  **Remained articles after the title/abstract screening: 1274** |

**Supplementary. Table 2.** Search strategy for left ventricular

| **Research question** | What are the clinical outcomes, prognosis, and imaging characteristics of the left ventricular aneurysm post-myocardial infarction? |
| --- | --- |
| **Research title** | Clinical characteristics and imaging findings in post-infarction left ventricular pseudo aneurysm and aneurysm, A pooled analysis of 21000 patients |
| **PICOT** | **P:** People with left ventricular aneurysm post-myocardial infarction  **E:** NA  **C:** NA  **O:** Clinical outcomes, Strategies of treatment, Prognosis, different imaging modalities characteristics  **T:** Observational studies |
| **Keywords** | Aneurysm, LVA, Myocardial infarction, Systematic review, Left ventricle rupture, Myocardial infarction complications, True aneurysm |
| **Search strategy**  (Searched on September 1^st^ 2022) | **Scopus:** ( ( TITLE-ABS-KEY ( "true aneurysm" ) ) OR ( TITLE-ABS-KEY ( "Ventricular aneurysm" ) ) ) AND ((("MYOCARDIAL INFARCTION") OR (MI) OR ("myocardial ischemia"))) AND ( ( TITLE-ABS-KEY (( "Cardiac magnetic resonance imaging" ) OR (" CMR") OR ( TITLE-ABS-KEY ( "Echocardiography" ) ) OR ( TITLE**-**ABS-KEY ( "Computed tomography" ) ) )  **Total citations that were retrieved from Scopus: 1090** |
|  | **PubMed:** ((((("Aneurysm"[Mesh]) OR ("Heart Aneurysm"[Mesh])) OR ("Ventricle aneurysm"[Title/Abstract])) OR ("True aneurysm"[Title/Abstract])) AND (("Myocardial Infarction"[Mesh]) OR ("Myocardial Ischemia"[Mesh]))) AND (((("Magnetic Resonance Imaging"[Mesh]) OR ("Tomography, X-Ray Computed"[Mesh])) OR ("Echocardiography"[Mesh])) OR ("Computed Tomography Angiography"[Mesh]))  **Total citations that were retrieved from PubMed: 1986** |
|  | **Google Scholar** Phrase searches: “left ventricular aneurysm”, “ventricular aneurysm”, “true aneurysm”, combined with “myocardial infarction”, “post‑MI”, “MI” |
| **Search Limits** | **Date range:** January 1991 – September 1, 2022 |
|  | **Rationale for start year:** 1991 corresponds to the earliest modern imaging‑confirmed LVPA cases meeting criteria |
|  | **Language:** English |
|  | **Included:** Case reports, case series, cohort studies |
|  | **Excluded:** Reviews, conference abstracts, animal studies |
| **Screening Results** | **Number of total citations that were retrieved after Scopus and PubMed systematic search and google scholar manual search: 3076**  **Remained articles after duplicates removal: 2593**  **Remained articles after the title/abstract screening: 581**  **The total number of included studies: 120 articles comprising 93 case reports and 27 case series and cohort studies** |

**Supplementary. Figure 1**. PRISMA flowchart diagram of study selection for pseudoaneurysm cases.

**Supplementary. Table 2**. Search strategy for left ventricular aneurysm.

Excluded Records
(n = 895)

## Identification

## Eligibility

Duplicated Records
(n = 1580)

Records identified through database searching
(n = 9210)

Full-text articles assessed for eligibility
(n = 1274)

Records screened by title/abstract: 7630
(n =783)

## Included

## Screening

Included Studies: 379

Case reports: n = 366

Case series: n= 13

Excluded Records
(n = 6356)

**Supplementary. Figure 2**. PRISMA flowchart diagram of study selection for true aneurysm cases.

Excluded Records
(n = 463)

## Identification

## Eligibility

Duplicated Records
(n = 483)

Records identified through database searching
(n = 3076)

Full-text articles assessed for eligibility
(n = 583)

Records screened by title/abstract: 2593
(n =783)

## Included

## Screening

Included Studies: 120

Case reports: n = 93

Case series: n= 27

Excluded Records
(n = 2012)

**Supplementary. Table 3:** Quality assessment of included studies.

| **Items** | **Atik et al.** | **Hui et al.** | **Figueras et al.** | **Hui et al.** | **Komeda et al.** | **Lafci et al.** | **Eren et al.** | **Pretre et al.** | **Moreno et al.** | **Narin et al.** | **Prifti et al.** | **Konen et al.** | **Purcaro et al.** |
| --- | --- | --- | --- | --- | --- | --- | --- | --- | --- | --- | --- | --- | --- |
| **Were there clear criteria for inclusion in the case series?** | + | + | + | + | + | + | + | + | + | + | + | + | + |
| **Way for all participants included in the case series?** | + | + | + | + | + | + | + | + | + | + | + | + | + |
| **Were valid methods used for the identification of the condition for all participants included in the case series?** | + | + | + | + | + | + | + | + | + | + | + | + | + |
| **Did the case series have consecutive inclusion of participants?** | + | + | + | + | + | _ | + | _ | + | + | + | + | + |
| **Did the case series have complete inclusion of participants?** | + | + | _ | + | + | _ | + | _ | _ | + | + | + | _ |
| **Was there clear reporting of the demographics of the participants in the study?** | + | + | + | + | + | + | + | + | + | + | + | + | + |
| **Was there clear reporting of clinical information of the participants?** | + | + | + | + | + | + | + | + | + | + | + | + | + |
| **Were the outcomes or follow-up results of cases clearly reported?** | + | + | + | + | + | + | + | + | + | + | + | + | + |
| **Was there clear reporting of the presenting site(s)/clinic(s) demographic information?** | + | + | + | + | _ | + | + | + | _ | + | + | _ | + |
| **Was statistical analysis appropriate?** | + | + | + | + | + | + | + | + | + | + | + | + | + |
| **Total score** | 10 | 10 | 9 | 10 | 9 | 8 | 10 | 8 | 8 | 10 | 10 | 9 | 9 |

| Supplementary. Table 4: Summary of case series and cohort studies. | | | | | | | | |
| --- | --- | --- | --- | --- | --- | --- | --- | --- |
| ID | Article | year | country | Study  type | N | Diagnostic imaging | | Surgical Treatment |
|  |  |  |  |  |  | Echo | CT/MRI/CXR |  |
| Pseudoaneurysm | | | | | | | | |
| 1 | Prifti et al. | 2017 | Albania | Case series | 13 | TTE:100%  TEE:69% | NR | 13/13 (100%) |
| 2 | Hui li et al. | 2009 | China | Case series | 7 | TTE:100% | CT: 28.5%  CXR:32.8% | 7/7 (100%) |
| 3 | Narin et al. | 2008 | Turkey | Case series | 5 | TTE:100% | MRI:42.9% | 5/5 (100%) |
| 4 | Atik et al. | 2007 | USA | Case series | 30 | TTE:72%  TEE:79% | MRI:6.7% | 30/30 (100%) |
| 5 | Eren et al. | 2007 | Turkey | Case series | 14 | TTE:57.1% | NR | 14/14 (100%) |
| 6 | Lafci et al. | 2006 | Turkey | Case series | 8 | TTE:100% | NR | 8/8 (100%) |
| 7 | Konen et al. | 2005 | Canada | Retrospective cohort | 4 | NR | MRI:100% | NR |
| 8 | Moreno et al. | 2002 | Spain | Case series | 6 | TTE:100% | NR | 1/7 (17%) |
| 9 | Figueras et al. | 2001 | Spain | Retrospective cohort | 8 | Doppler:100% | NR | NR |
| 10 | Pretre et al. | 2000 | Switzerland | Case series | 7 | TTE:100% | NR | 7/7 (100%) |
| 11 | Purcaro et al. | 1997 | Italy | Case series | 28 | TTE:100%  Doppler: 100% | NR | 24/28 (85.7%) |
| 13 | Komedo et al. | 1992 | Canada | Case series | 12 | TTE: 50%  Doppler: 25% | NR | 12/12 (100%) |
| 14-379 | Pooled case reports | 1991-2022 |  |  | 366 | TTE:73.2%  TEE:10.9% | MRI:23.2%  CT:31.1%  CXR:24.8% | 449/479 (95.5%) |
| Aneurysm | | | | | | | | |
| 1 | Chen et al. | 2022 | China |  | 92 |  |  |  |
| 2 | You et al. | 2021 | China | Prospective cohort | 150 | TTE:100% | NR | NR |
| 3 | Ning et al. | 2018 | China | Retrospective cohort | 575 | TTE:100% | NR | NR |
| 4 | Vallabhajosyula et al. | 2020 | USA | Retrospective cohort | 17626 | NR | NR | NR |
| 5 | Yan et al. | 2018 | China | Retrospective cohort | 102 | TTE:100% | NR | 102/102  (100%) |
| 6 | Haranal et al. | 2017 | India | Retrospective cohort | 25 | TTE:100% | CXR:100% | 25/25  (100%) |
| 7 | Pang et al. | 2015 | China | Retrospective cohort | 125 | NR | NR | NR |
| 8 | Taha et al. | 2013 | Iraq | Case series | 27 | TTE:92.6% | NR | 27/27  (100%) |
| 9 | Chen et al. | 2012 | China | Retrospective cohort | 194 | TTE:100% | NR | 194/194  (100%) |
| 10 | Wang et al. | 2012 | China | Retrospective cohort | 62 | TTE:100% | NR | 62/62  (100%) |
| 11 | Haung et al. | 2011 | China | Retrospective | 248 | TTE:100% | NR | 248/248  (100%) |
| 12 | Marsan et al. | 2010 | Netherland | Prospective cohort | 52 | TTE:100% | MRI:100% | NR |
| 13 | Coskun et al. | 2009 | Germany | Retrospective cohort | 269 | TTE:100% | NR | 269/269  (100%) |
| 14 | Chen et al. | 2007 | Taiwan | Case series | 9 | TTE:100% | NR | 9/9  (100%) |
| 15 | Mukadirov et al. | 2007 | France | Retrospective cohort | 53 | TTE:100% | NR | 53/53  (100%) |
| 16 | Yildirim et al. | 2007 | Turkey | Retrospective cohort | 18 | NR | NR | NR |
| 17 | Tappainer et al. | 2007 | Italy | Case series | 12 | TTE:100% | NR | 12/12  (100%) |
| 18 | Lange et al. | 2005 | Germany | Retrospective cohort | 305 | NR | NR | 305/305  (100%) |
| 19 | Lundblad et al. | 2004 | Norway | Retrospective cohort | 159 | TTE:86.1% | NR | 159/159  (100%) |
| 20 | Antunes et al. | 2004 | Portugal | Retrospective cohort | 110 | TTE:100% | NR | 110/110  (100%) |
| 21 | Trehan et al. | 2003 | India | Retrospective cohort | 129 | TTE:100% | NR | 129/129  (100%) |
| 22 | Mickleborough et al. | 2001 | Canada | Retrospective cohort | 193 | TTE:29.5% | NR | 193/193  (100%) |
| 23 | Cherniavsky et al. | 2001 | Russia | Retrospective cohort | 41 | TTE:100% | NR | 41/41  (100%) |
| 24 | Castiglioni et al. | 2001 | Italy | Retrospective cohort | 94 | TTE:100% | NR | 94/94  (100%) |
| 25 | Shapira et al. | 1997 | USA | Retrospective cohort | 47 | TTE:57.4 | NR | 47/47  (100%) |
| 26 | Sinatra et al. | 1997 | Italy | Retrospective cohort | 118 | TTE:100% | NR | 118/118  (100%) |
| 27 | Donato et al. | 1995 | Italy | Case series | 62 | NR | NR | 62/62  (100%) |
| 28-119 | Pooled case reports | 1991-2022 |  |  | 91 | TTE:75.8%  TEE:8.8% | MRI:24.1%  CT:18.7%  CXR:26.3% | 56/91  (61.55%) |

CT, computed tomography; CXR, chest x ray; MRI, magnetic resonance imaging; NR, not reported; TEE, trans esophageal echocardiography; TTE, trans thoracic echocardiography
